# Exploring caseworkers’ experiences of SMILE: Facilitating sustainable employment for people with complex needs

**DOI:** 10.1177/22799036261456001

**Published:** 2026-06-12

**Authors:** Simon Asplund, Magnus Svartengren, Teresia Nyman, Åsa Fichtel, Therese Hellman

**Affiliations:** 1 Department of Medical Sciences, Occupational and Environmental Medicine, 214437 Uppsala University, Uppsala, Sweden; 2 Department of Occupational and Environmental Medicine, Uppsala University Hospital, Uppsala, Sweden

**Keywords:** complex needs, unemployment, SMILE, work integration, person-centred support, caseworker

## Abstract

**Introduction:**

Research suggests that holistic, person-centred support may be effective in facilitating employment for people with complex needs. A model based on such principles, labelled SMILE, was developed for this purpose. This study aimed to explore caseworkers’ experiences using SMILE, to identify which aspects they perceive as key to helping people with complex needs achieve sustainable employment.

**Design and methods:**

The study employed a qualitative approach, using semi-structured interviews with 13 caseworkers who had first-hand experience working with the model to enhance employability among people with complex needs. Data were analysed using thematic analysis.

**Results:**

The respondents emphasised the model’s holistic, person-centred support as essential for enabling targeted and relevant interventions. They also stressed the importance of clients having access to all interventions through a single point of entry. They also highlighted that progress served to energise and motivate clients. Another key aspect was ensuring the availability of continuous and easily accessible support from caseworkers, providing a sense of security for clients. This allowed them to make progress, cultivating empowerment and leading to a sense of self-efficacy and belief in their ability to attain sustainable employment.

**Conclusions:**

Key aspects of the model that facilitated employment for people with complex needs included: providing holistic, person-centred support accessible through a single access point. Continuous and easily accessible support from a consistent caseworker was also essential for facilitating steady progress and fostering a sense of self-worth and confidence in their ability to gain employment.

## Introduction

Unemployment is an important public health concern. Previous research has shown that it is associated with both mental illness and poor physical health.^1–3^ One group particularly disadvantaged in the labour market is people with complex needs – those suffering from various health-related, cognitive and/or social challenges that are often interconnected.^4,5^ These individuals face many barriers to achieving sustainable employment and financial independence, which can lead to health inequalities.^6,7^ In contrast, labour market participation is associated with several perceived benefits,^8–10^ and is considered a fundamental social determinant of good health. This highlights the importance of establishing well-functioning interventions to support people with complex needs to integrate into the labour market.

Research on interventions specifically supporting people with complex needs into working life is limited. However, several important factors have been highlighted in promoting labour market participation among individuals with mental illness. A qualitative study emphasised the need for a holistic, person-centred approach, involving professionals who instil hope and demonstrate confidence in their clients’ potential,^11^ an approach also valued by those receiving support.^12^ Previous research has shown that supported employment (SE), a vocational rehabilitation intervention with emphasis on empowerment and the explicit aim of facilitating competitive employment, can be effective for increasing labour market participation among individuals with disabilities.^13,14^ For instance, the Individual Placement and Support (IPS) model has shown positive results for individuals with mental illness,^15^ as well as for young adults with reduced working-capacity due to various social or health-related issues.^16^ IPS also appears to have a long-term positive impact on labour market participation among those with mental health concerns,^17–19^ highlighting that individually tailored support may be an effective solution for those struggling to enter the labour market.

Supported employment has been found to be effective for people with complex needs, especially when it is individually tailored and emphasises user participation and choice.^5^ Collaborative strategies that combine vocational training and therapeutic support, while actively involving end users, have also been found effective.^20^ Dew et al.^21^ highlight the principles of autonomy, agency, choice, and control for those with complex needs, arguing that these principles foster a sense of self which is necessary for their ability to identify aspirations, develop skills and achieve goals. This aligns with self-efficacy theory,^22^ which emphasises the role of individual agency and the belief in one’s own capacity to act in order to achieve desired outcomes. A strong sense of self-efficacy supports motivation, well-being, and personal accomplishment, all of which are essential for goal attainment, and has been argued to be a promising predictor of vocational outcomes for individuals with mental health issues.^23–25^

Rankin and Regan^4^ suggest that people with complex needs often fall through the cracks in service provision, emphasising the necessity for service managers to adopt a holistic approach when working with this group. This approach should include, for instance, addressing multiple needs simultaneously, promoting user engagement and empowerment, and providing access to a range of supportive interventions through a single point of entry. They advocate for appointing “service navigators” who can assist individuals in navigating the complexities of health, social care, housing, and employment services. Gore et al.^26^ also highlight the central role of caseworkers in facilitating employment for people with complex needs, explaining that it can be a challenging process and underlining the importance of allocating sufficient time and resources to help clients achieve their goals. They stress the need for inter-organisational collaboration to improve employment outcomes for this target group, while also advocating for enhancing service-user empowerment, underlining the importance of working “with” clients rather than “for” them. Similarly, research on frontline workers experiences of welfare-to-work services for young adults with complex needs show that administrative and organisational barriers often hinder cross-sectoral collaboration that might enhance labour market opportunities for this group.^27^ Similar to “service navigators”, the authors argues for the importance of boundary spanners that can help integrate various services and coordinate efforts for people with complex needs.^27^ In Sweden, people with complex needs often face fragmented services and difficulties in comprehending and navigating the “system”.^28^ This further highlights the potential key role of caseworkers in bridging the gap between organisations, enabling easier access to various services and labour market opportunities for people with complex needs.

A recent Danish project examined factors that facilitate people with complex needs in achieving sustainable employment. The study found that progress toward labour market integration was highest among those engaged in parallel activities, such as skill or competence development, and various social or health-related interventions. Consistent support from the same caseworker also improved employment outcomes, as job prospects declined significantly with each change in caseworker.^29^ The results from this project were later adapted into a model and working method for the Swedish context, with the aim to strengthen labour marker participation for individuals with complex needs. This model and working method is labelled SMILE (The Scaling Model for Individual Learning and Empowerment).^30^

SMILE is theoretically grounded and based on five core principles and seven indicators (Table 1). These aspects have been identified as important for work integration in previous research.^11,12,30^ However, these core principles have not been empirically evaluated, for example in a programme theory evaluation,^31^ and thus there is a lack of knowledge regarding how the model is perceived by those delivering and receiving an intervention based on this model. By exploring the experiences of caseworkers, we hope to gain further insight into the model and its inherent mechanisms, in order to understand which aspects are of importance and how they might facilitate employment outcomes for this group. Thus, this study aimed to explore caseworkers’ experiences using SMILE, to identify which aspects they perceive as key to helping people with complex needs achieve sustainable employment.

**Table 1.** List of five core principles and seven indicators of SMILE.

Core principles	Indicators
Confidence in the individual’s ability	Managing daily life
Caseworker continuity	Health management
Parallel activities	Job search behaviour
Continuous activities	Belief in getting a job
Focus on getting a job	Knowledge of the labour market
​	Determination
​	Cooperation skills

## Design and methods

This qualitative study was based on semi-structured interviews with caseworkers who had first-hand experience working with SMILE. The accounts presented by the caseworkers in this study are discussed and compared with the theoretically grounded core principals of SMILE, in order to assess which aspects of the model that are key in municipal labour market support for people with complex needs, as well as how the intended mechanisms are evident in practice.

Ethical approval for this study was obtained from the Swedish Ethical Review Authority (Dnr 2024-04251-01).

## The Scaling Model for Individual Learning and Empowerment (SMILE)

SMILE aims to strengthen labour market participation among those requiring additional support to enter working life. The model and working method are described in a manual including information on core principles which can be seen as important prerequisites for successful work with the method. Furthermore, the manual contains information about the working method focusing on dialogue about seven indicators (see table 1). The manual also incorporates various assessment tools and questions that can be used in structured dialogue-sessions between client and caseworker.

The structured dialogue between the caseworker and the individual (hereinafter referred to as client) occurs every three months, focusing on seven indicators that are important for a person to attain employment or enter studies. These include managing daily life, health management, job search behaviour, belief in getting a job, knowledge of the labour market, determination, and cooperation skills.

The first dialogue-session with the client ends with the creation of an activity plan, which is then continuously followed up over time. Structured follow-up sessions are typically held every three months to evaluate the client’s needs and progress. Between these sessions, clients meet regularly with their caseworker to discuss their situation, health, progress, and current needs, receiving support connected to various aspects of their life. SMILE provides a professional basis for assessment, where the dialogue between the individual and caseworker concentrates on various areas that are important for employment or education.^30^ The model is not an intervention in and of itself, but offers structured materials, methods, and attitudes to evaluate client needs and coordinate targeted interventions, helping to systematise and facilitate the process toward employment.

Initially, caseworkers undergo a one-day training-programme in the core principles and practical implementation of the model and working method, after which they begin adhering to these principles and using the manual together with their clients.

## Study context

SMILE is used in departments within municipalities aimed at enhancing employability among people with complex needs. These departments offer various interventions to help those requiring additional support to gain work experience through vocational training, but they also arrange group activities to strengthen social and collaborative skills, as well as supporting clients with everyday life tasks. This includes helping clients access appropriate help from other authorities, such as the healthcare system or the Swedish Social Insurance Agency. It also includes helping clients with issues related to private economy or housing situation, or other areas in which they struggle and need assistance. Nevertheless, the primary goal is for clients to eventually secure employment or pursue studies.

Eligibility criteria for support from these departments varied somewhat between the municipalities, but all clients were unemployed, received financial aid, and had various social, cognitive, or health-related issues. They were assessed as requiring more extensive vocational rehabilitation and interventions than what was typically provided through the standard employment preparation services of the Swedish Public Employment Agency. Consequently, they were referred to these departments, where caseworkers specifically trained in delivering targeted support to people with complex needs used SMILE as an integral part of their practice. Clients often had multiple supportive interventions in place, and caseworkers working in the departments frequently collaborated with other authorities to ensure coordinated support. While the frequency of contact varied depending on clients’ involvement in other vocational activities, they maintained regular contact with their assigned caseworker from enrolment in the department until discharge, which typically occurred upon securing employment or engaging studies. In this context, SMILE was used as an approach both related to how caseworkers should work with and behave toward clients, but also as a tool for continuously assessing clients’ situations, evaluating the impact of interventions, and planning for future actions to improve employability.

## Data collection and participants

For this study, a purposive sampling was used^32,33^ to include individuals with first-hand experience using SMILE. The decision to interview caseworkers was driven by their comprehensive understanding of the model and how it supports people with complex needs in securing employment. During the recruitment phase, managers from municipal departments using SMILE were contacted. These managers then contacted caseworkers who had previously undergone training in the model, assessing their interest in participating in the study. In total, 14 participants from four different municipalities were identified and contacted via email, which included written information about the study. One participant later dropped out, which left a total sample of 13 participants from four municipalities. The municipalities varied in population size and geographical location and were selected to provide a more comprehensive and representative understanding of the model, while accounting for local differences in implementation. All 13 participants had similar roles, working as caseworkers assigned to support people with complex needs seeking employment or further education. The mean age of participants was 43 years (range 26-62). Seven participants worked in smaller communities, and four were based in a large city in Sweden. The sample comprised two men and 11 women. The somewhat disproportionate gender distribution was due to women being overrepresented within the profession.

All interviews were conducted at the participants’ workplaces during the fall of 2024 by a research assistant. Each participant were informed about the aim of the study and signed a written consent form before the start of each interview. The interviews were conducted face-to-face, lasting approximately 45 minutes (range 35-64). A semi-structured interview guide was employed to ensure the interviewer remained focused on the research topic while also allowing flexibility to explore relevant issues that emerged during the conversations.^34^ During the interviews, participants were asked questions about various aspects of the model, including its strength and weaknesses, how they used it in their daily work, while sharing experiences from particular cases. The interview guide was developed together by all authors, and the questions were tailored to encompass all core principles of the model,^30^ to ensure that important aspects were not overlooked. The first interview was seen as pilot-testing of the interview guide, after which minor revisions were made in terms of a few additional questions. Still, the minor revisions allowed for inclusion of this first interview. All interviews were digitally recorded and transcribed verbatim using Kahubi, a transcription software. Thereafter, the researchers listened to and checked the audio recordings to correct any inaccurate phrasings. The quotes used in the results section were later translated into English by one of the authors.

## Data analysis

Data were analysed using thematic analysis, inspired by the six steps presented by Braun and Clarke.^35^ Initially, all transcripts were repeatedly read in order to gain a comprehensive understanding of the material. The material was then imported into Nvivo 14 for further analysis, where initial codes based on semantic content were generated. For the first interview being coded, a thorough discussion about how the coding had been carried out followed between the first and last author. As the basic coding was found to be consistent, the first author coded all transcripts in the same way and the procedure was verified by last author on regular occasions to improve credibility. The codes were thereafter collated into preliminary categories reflecting latent meaning within each transcript. These categories were then analysed across transcripts and merged into broader themes and sub-themes representing common patterns relevant to the research topic. The next phase of the analysis involved reviewing and refining the themes by revisiting the transcripts and coded extracts while considering how they fit each theme where they were placed. During this phase, the themes were thoroughly discussed among the authors to ensure credibility and consensus regarding the interpreted meanings. The feedback from the authors resulted in some of the themes being slightly adjusted to further enhance the latent content and better align with the aim of the study. This process continued until a consensus was reached, resulting in the final themes: “Enabling a holistic view of the individual” and “Cultivating empowerment through continuity and accessible caseworker support,” along with their corresponding sub-themes (see Table 2). Finally, using the gathered data, the final report was developed, and quotes from the transcripts were used to further enhance transparency and demonstrate the prevalence of each theme.^35^ Continuous discussions among the authors were held during this phase, with transcripts reviewed collaboratively to ensure a consistent interpretation of the data and its inherent meanings. This collaboration provided an interdisciplinary approach as the research group includes medical doctor, psychologist, occupational therapist, physiotherapist and sociologist.

**Table 2.** Themes and sub-themes.

Themes	Sub-Themes
Enabling a holistic view of the individual	Enabling targeted and relevant supportive interventions A focal point for other interventions and collaboration Highlighting progress to energise and motivate clients
Cultivating empowerment through continuity and accessible caseworker support	Enabling continuity and a sense of security Facilitating a sense of self-efficacy

## Results

### Enabling a holistic view of the individual

#### Enabling targeted and relevant supportive interventions

One key strength of SMILE was its ability to systematically assess and identify clients’ needs, conditions, and preferences using the model’s assessment tool. This process helped map out strengths and, more importantly, areas requiring improvement. The mapping was important to enable targeted and relevant interventions, facilitating clients’ development and improvement in areas where they displayed deficiencies. Several caseworkers indicated that clients’ responses provided an effective basis for discussions regarding various issues and possible solutions going forward. Although gaining employment was the long-term objective, clients often faced a range of challenges that needed to be addressed before they were ready to enter working life – challenges that the material helped to illuminate. SMILE offered a structured approach to this process, offering extensive materials that caseworkers could rely upon for guidance. This helped organise and render the process more transparent for clients. Several caseworkers explained that the material allowed clients to reflect on and articulate how they perceived themselves while identifying potential areas for improvement.

Well, I feel that… It's a good form. A questionnaire to start from. The fact that it’s… that it's, like, the individual themselves who gets to… who gets to answer how they see themselves. And that it provides a good, like, basis for discussion for figuring out. But what are your needs, you know? What are your strengths and weaknesses? I think it's very good. (Respondent 3)

The systematic assessment of various areas enabled clients to voice their thoughts and opinions about their own situation and preferences going forward, creating a foundation for discussions regarding what type of support or interventions they would need going forward. Almost all caseworkers acknowledged that the material was effective for evaluating clients; however, occasionally, they needed to adjust their approach or modify the questions, as some clients struggled to comprehend certain phrasings or formulations. One caseworker explained how she occasionally uses visual aids from the material to better identify clients’ conditions and needs.

We use, as a starter kit, this form during our first meeting with new participants, where they rate themselves with the questions and can read about the different parts of the assessment. But sometimes, it has proven to be a bit difficult for some participants. Then, we’ve used these parts where there is a smiley face and a bit like that to help facilitate the conversation. It allows us to talk more about it, because you might get stuck on the wording and then it becomes an easier way sometimes to be able to follow up. (Respondent 1)

Another caseworker, who also voiced concerns about the occasionally abstract nature of the questions, stated that the material does provide suggestions and guidance on how to phrase questions to be more accessible and comprehensible for clients. However, a few caseworkers reported uncertainty regarding how to support clients experiencing more severe mental health difficulties, emphasising that the material lacked adequate guidance or practical strategies for addressing these complex situations. Caseworkers also used the questions and assessment tool to varying degrees; however, most of them agreed that the material was effective for mapping clients, enabling a coherent trajectory where they could plan appropriate and relevant supportive actions.

#### A focal point for other interventions and collaboration

One primary contribution of SMILE was that it provided a focal point for the array of activities and supportive actions surrounding clients, providing organisation and coordination of the various components needed for an effective transition into working life. The caseworkers highlighted that clients typically engage in multiple parallel supportive activities – a key feature of SMILE – yet require assistance in keeping track of, and coordinating, these efforts.

Collaboration around the people, we’ve seen, is very successful […] Because what you often experience when meeting people is that they have different interventions surrounding them, but they don't really know what they are and why, or what they’re about, and some work this way and some work that way. (Respondent 8)

The caseworkers emphasised the significance of networking and collaboration in addressing clients’ various needs. For instance, the majority of clients were in poor health, often suffering from problems such as depression, anxiety, social isolation, and/or other mental health-related concerns. A common factor was that many had either undiagnosed medical problems, had been on medical leave, or had limited social engagement, staying primarily at home. Thus, this aspect was often strongly linked to the type of interventions needed and had to be addressed. Interventions often involved therapy, medication, housing- or financial assistance, or engagement with occupational therapists or rehabilitation coordinators. Several caseworkers stressed that parallel health-interventions were both common and often crucial for preparing clients to enter working life, and that SMILE provided a centralised location for coordinating all these efforts. Some characterised their role as a “spider in the web,” connecting all relevant contacts to improve clients’ access to available resources and helping them maintain their appointments and commitments.

Yes, my task is – well, the long-term goal is to help people into employment or studies. And support these individuals with coordination, with contacts and make sure that it all flows smoothly. And that they get the support and interventions they need. It's a bit like being the spider in the web for the participant. (Respondent 9)

Moreover, the caseworkers stressed the importance of clients engaging in practical activities, such as vocational training and/or other group activities, in order to learn how to navigate various social contexts. Here, SMILE served to connect and align other interventions and activities for the clients. The assessments conducted in between were shaped by the outcomes and experiences of these activities, enabling clients and their caseworkers to engage in discussions about the broader context and how these activities contributed to the overall process of achieving employment. Such contributions could include learning to follow schedules and routines, maintain regular attendance, and collaborating with others. Thus, the role of SMILE was to assess and highlight the impact of these interventions in preparing clients for the next step toward entering working life.

#### Highlighting progress to energise and motivate clients

SMILE provided an effective way to track clients’ progress over time, helping to energise and motivate them. By utilising the systematic assessment embedded in the material during follow-up coaching sessions, clients were able to observe their development and clearly identify areas where they had improved.

Yes. But when you've done it several times, used this material, and you see that it's moving forward, like the numbers changing, and how they rate themselves differently and so on. Then I’ve felt that it gives them some energy. It is important for them to see. It’s not just that you talk about it… Now you can really show them. ‘This is how you answered last time, and this is how you are answering now. (Respondent 3)

This was a seemingly significant aspect of SMILE, as one caseworker described, “We do not work with employment; rather, we work solely with progress” (respondent 6). Another caseworker described how clients gain a clear understanding of their progress, revealing the positive outcomes of their efforts and emphasising that SMILE has played a pivotal role in this process.

I actually think it has more of an impact on the person too. Particularly with the person I told you about earlier – you could really see it. The first SMILE assessment was very low, and then by the second one, there had been a change, and it became so clear for the participant. (Respondent 8)

The caseworkers described this as making clients’ development clear and visible, highlighting that observing progress cultivates a sense of empowerment, fostering motivation, and strengthening clients’ belief in their ability to achieve sustainable employment. It was also beneficial to discuss the factors that contributed to improvement, helping clients identify positive behaviours or efforts that could further enhance their self-efficacy. However, occasionally, clients would score lower than in previous assessments, indicating a regression in development. In such instances, SMILE helped reveal areas where progress was lacking and where further efforts were needed.

Another key aspect in helping clients manage their progress and stay motivated was the caseworkers’ role in ensuring they engaged in continuous ongoing activities and coaching sessions, to prevent disruptions in the process. The caseworkers explained that clients can enter a state of flow during which meaningful progress occurs, allowing them to develop and recognise their own capabilities. However, if consistency in coaching sessions and/or activities was disrupted, they often lost focus and became disengaged from their objectives.

I think continuous efforts are very important, because you can clearly see that as soon as there’s a gap or they don’t have anything going on, people often lose their progress, so I think it’s very important. (Respondent 11)

### Cultivating empowerment through continuity and accessible caseworker support

#### Enabling continuity and a sense of security

SMILE ensured that clients were supported by the same caseworker throughout the entire process leading up to employment and/or studies, providing continuity and stability. The caseworkers emphasised the vulnerability of their clients and the importance of having access to a reliable person for support whenever challenges arise or when guidance is needed. They expressed that clients need someone they can trust, and that establishing such a relationship takes time. This highlights the necessity of remaining with the same caseworker rather than being frequently reassigned. The caseworkers also described how many clients experience difficulties engaging in new social connections, thus being grateful for receiving continuity with the assigned caseworker.

*It's clear that they appreciate this continuity because we’re working with a challenging group, and they find it very difficult. Most of them, not all, of course, find it hard to make new contacts and it takes time to build trust. So it's very important to be there throughout the* whole *process. I see a real benefit in that. And I think they’re very grateful for it too.* (Respondent 5)

Through caseworker continuity, SMILE provided a much-needed sense of security for clients when (re)integrating into working life. This sense of security empowered clients and helped them to develop the courage to engage in activities and/or seek help for various health-related issues. Clients were described as requiring the presence of someone by their side who can make things possible, provide support, and offer encouragement and motivation. SMILE facilitated this by ensuring caseworkers remained consistently engaged with the same clients throughout the entire process. One caseworker explained that when clients are offered continuity, they become more willing to disclose issues they had previously kept private, enabling further discussion of possible solutions and necessary interventions going forward. When continuity in support was lacking, or when clients for various reasons (e.g., holidays, employee turnovers, etc.) had to switch to another caseworker, it often led to a significant decrease in progress and perseverance, negatively affecting their chances of securing employment.

Furthermore, caseworker continuity also aided in facilitating smooth transitions for clients, for instance, when entering working life, by providing continued security as they adjusted to their new situation.

Here, you get continuity, and we’ve noticed that it’s very important for us to remain involved when someone starts working or studying, so that they can cope with and maintain it until it becomes a true source of stability, a new everyday life they can settle into. But they can also just give me a call or send a text message or email. Then we can just have a conversation. (Respondent 2)

Besides providing emotional support for clients transitioning into their new situations, the caseworkers also stressed the importance of ensuring that integration into the workplace proceeds smoothly for employers, working collaboratively to adjust conditions if needed. However, transitional support was not limited to clients’ integration into the workplace, as caseworkers also accompanied clients to vocational training, medical appointments, etc., offering a sense of security in all new contexts involving unfamiliar individuals.

#### Facilitating a sense of self-efficacy

Another key aspect of SMILE was its ability to help clients build self-confidence and develop a sense of self-efficacy, fostering trust in their own ability to achieve sustainable employment. Attaining self-efficacy could be seen as the result of the collective impact of all elements of SMILE. Such aspects involved taking a comprehensive approach to each client by evaluating specific obstacles, helping to plan appropriate and achievable activities, and promoting steady progress while making this visible to the clients. As clients gradually gained more stability, they started to recognise their potential and see entering working life as a real possibility. Over time, and with continuous support from their caseworkers, they developed a renewed sense of self-efficacy, leading to the confidence and resilience necessary to pursue sustainable employment. However, this was an issue that required ongoing attention from caseworkers, as low self-esteem and a lack of self-confidence were common challenges among those receiving support. As a fundamental indicator of SMILE, the development of self-belief was regarded as essential to clients’ success and a key responsibility of the caseworkers to foster.

Maybe it’s simply the individual's belief in their ability to take on a job [that’s the most important factor], that they dare to believe in themselves. Because if they don't, then it's an uphill battle. And I think that's part of my role too, to coach them in a way that helps strengthen their belief in themselves. (Respondent 6)

The caseworkers emphasised that it is their responsibility to help clients identify their personal strengths, continually reinforcing and nurturing their belief in their own capabilities. For instance, through structured planning, they could deconstruct the process into smaller, more manageable components, promoting a sense of achievement and preventing clients from feeling overwhelmed. They explained that even small steps can initially seem daunting, emphasising the importance of a gradual, tailored approach to exposure considering each client’s circumstances.

And sometimes you can talk about how it feels like you're standing in front of a big mountain or a long road, as if it's impossible to get where you want to go. Then you can talk about the idea of maybe going around it. You don't have to go over all that or go straight through the road, but you can take small steps around it instead. And that works well too– that you have to like break down all these overwhelming things into smaller, manageable things. (Respondent 2)

Furthermore, a fundamental aspect in facilitating self-efficacy among clients was having the same caseworker throughout the process. This continuity allowed caseworkers to reflect with clients on their previous situations in comparison to their current circumstances. With a comprehensive understanding of each client’s progression, caseworkers could highlight this distinction, enabling a reflection that served to illuminate the significant changes that had occurred. Such discussions were considered important, as they helped clients recognise their own development, empowering them and reinforcing the belief that they were capable of finding employment.

## Discussion

This aim of this study was to explore caseworkers’ experiences using SMILE, to identify which aspects they perceive as key in helping people with complex needs achieve sustainable employment. This was explored through semi-structured interviews with an interview guide based on the core principles of SMILE and thus allowed for a comprehensive examination of the theoretical foundation. The findings showed that the caseworkers experienced the core principles as being important in order to strengthen labour market participation among individuals with complex needs. One aspect was the importance of maintaining a holistic perspective on individuals receiving support. SMILE facilitated this by helping assess various individual needs, conditions, and preferences, enabling the implementation of targeted and relevant supportive interventions. This holistic, individually tailored approach shares characteristics with IPS and aligns with previous findings on interventions for individuals with mental illness and various social and health-related problems.^11,12,16^ It is also relevant for people with complex needs, specifically.^4,5,21^ As Dew et al.^21^ highlight the importance of autonomy, agency, control, and choice, SMILE allows individuals to express their own voices, ensuring that their concerns, preferences, and needs are taken into account when planning the best possible actions going forward. This also aligns with the findings of Weston,^5^ emphasising user participation and choice, as well as with caseworker experiences described by Gore et al.,^26^ highlighting the importance of a collaborative approach and emphasising service-user empowerment. One way in which the model enhanced service-user empowerment was by providing a systematic and effective way of tracking individual progress, enabling clear observations of development, which led to clients feeling energised and motivated to continue their efforts toward securing employment.

Furthermore, previous studies have shown an association between unemployment and poor physical and mental health,^1–3^ with some suggesting that unemployment is not only associated with mental illness but may actually be a cause of mental distress.^36^ Those who received support from the caseworkers in this study faced various social- and health-related issues that needed to be addressed in order for them to become ready to enter working life. One key aspect of SMILE was that it served as a focal point for an array of interventions surrounding clients, such as vocational training and various social or health-related interventions. In this process, the caseworkers acted as centralised coordinators, collaborating with external organisations and organising all efforts on behalf of the clients. The caseworkers adopted a role similar to “service navigators” proposed by Rankin and Regan,^4^ addressing multiple needs simultaneously while helping clients navigate the complexities of various services and providing access to a range of supportive interventions through a single access point. This aspect was emphasised as fundamental to clients achieving their vocational goals, as they often struggled to keep track of and access all types of interventions on their own. As highlighted by previous research,^26^ several of the caseworkers mentioned the importance of maintaining good relationships with, and collaborating with, external organisations to facilitate the best possible vocational outcomes for people with complex needs.

Another significant finding from this study was the critical role of accessible and continuous caseworker support. Clients appeared to become progressively empowered as a result of receiving consistent support from the same individual throughout the entire process – a key feature of SMILE. Continuity and stability fostered trust between the client and the caseworker, promoting a sense of security. This ongoing support, both practical and emotional, was integral to clients’ development and increased confidence in their ability to secure employment, as they successfully managed tasks and activities they had previously struggled with. These findings are consistent with previous research,^11,12^ which emphasises the importance of clients being supported by professionals who show hope and maintain positive attitudes. These professionals provide person-centred support and believe in the clients’ ability to attain employment. This is particularly important because clients often struggle to view employment as a realistic goal for themselves. Additionally, the results also align with previous findings highlighting the necessity of allocating adequate time and resources to help clients achieve their employment goals.^12,26^ In this study, caseworkers noted that building a trusting relationship—a critical element for success—requires both time and effort, especially since forming new social connections was a challenging process for the clients.

Similarly, another central feature of SMILE was its ability to foster a sense of self-efficacy among clients, enhancing their belief in their ability to successfully participate in the labour market. This sense of self-efficacy did not emerge immediately; rather, it seemed to develop gradually through the cumulative influence of various components of the model, such as individually tailored, continuous, caseworker support, gradual exposure to activities, and the promotion of steady progress. Each of these elements contributed to the broader process of building clients’ confidence in their ability to attain sustainable employment. Previous research has shown an association between higher levels of self-efficacy and an increased likelihood of achieving vocational goals,^25^ and specifically, work-related self-efficacy with achieving vocational outcomes for individuals with mental health issues.^23,24^ Job-seeking, or vocational, self-efficacy has been linked to both social- and independence skills, and the ability to connect with others and adapt to one’s disability.^37,38^ Several caseworkers described how clients often initially struggled socially, but that gradual exposure to social activities helped them adapt to social situations and work together with others. This supports the notion of providing continuous and easily accessible vocational support, where clients can gradually become comfortable interacting with others, thereby boosting their confidence.

In summary, the theoretical core principles of SMILE are empirically supported as the caseworkers experienced that all principles may enhance labour market opportunities for people with complex needs. The core principle least salient during the interviews was focus on getting a job, although all participants agreed this was an important aspect. Furthermore, one limitation highlighted by the caseworkers was the labelling of certain questions in the manual. Some were perceived as overly abstract or difficult for clients to comprehend. This resulted in caseworkers occasionally needing to adapt their approach, either by rephrasing questions or, in some instances, omitting them entirely. Consequently, the various assessment tools and questions presented in the manual could benefit from incorporating a broader range of examples on how to phrase questions more effectively or in a way that is easier to understand. Additionally, some caseworkers noted the challenge of addressing clients’ diverse (mental) health issues. To address this, the model could be enhanced by offering more detailed, context-specific guidelines to help caseworkers navigate a variety of client situations, ensuring they are equipped to take appropriate and targeted actions.

## Methodological considerations and limitations

This study employed thematic analysis to explore caseworkers’ experiences using SMILE, to identify which aspects they perceive as key in helping people with complex needs achieve sustainable employment. Although the study sample consisted of only 13 participants, it was considered sufficient for the aim in terms of information power.^39^ When discussing the analytical process among the authors, it was deemed that data saturation was reached for this study in terms of covering all aspects of the model. Gathering various perspectives by incorporating participants from four different municipalities was fundamental in gaining a comprehensive and more representative understanding of the important aspects of the model, while accounting for differences in implementation at the local level.

Furthermore, although SMILE was generally positively perceived and valued as an effective tool by the participants, the results of this study cannot validate whether the model expedites the process of achieving sustainable employment for clients. Instead, the results highlight the core principles and mechanisms of the model and working method that caseworkers agreed were important for facilitating employment outcomes for people with complex needs, as well as why they were important and how they manifested in practice.

We also acknowledge the limitation of focusing solely on caseworkers’ experiences with using the model, without including people with complex needs who are the recipients of the support. However, a separate longitudinal study is currently underway, which involves conducting three interviews over a nine-month period with those who have received municipal support through the model, some of whom have since gained employment. By collecting data from both caseworkers and recipients, we aim to address potential critiques of a one-sided perspective, thus enabling a more comprehensive understanding of the model’s key components, as well as its potential limitations.

## Conclusions

Several key aspects of SMILE were emphasised as important in facilitating sustainable employment for people with complex needs. The findings suggest that clients benefit from holistic and person-centred support that simultaneously addresses multiple aspects of their lives to improve employability. Interprofessional collaboration and coordination, ensuring that various services are accessible through a single point of contact, also seemed important for becoming ready to enter working life. Furthermore, continuous and easily accessible support from a consistent professional emerged as a key factor in facilitating clients’ steady progress, while also fostering a sense of self-worth and confidence in their own abilities.

## Significance for public health

Unemployment is an important concern that impacts public health. People with complex needs often face multiple interrelated barriers to employment, which are linked to broader social determinants of health, such as social inclusion, economic stability, and access to coordinated care. The findings of this article have practical importance, as they emphasise the need for services that offer individualised support, combining vocational training with other types of psychosocial assistance. By ensuring access to consistent, person-centred support, such services can contribute not only to improving employability among marginalised groups but also to social inclusion and enhanced well-being for those with mental health concerns. These findings can inform policymakers and practitioners working to facilitate employment for individuals suffering from various health-related and/or social problems.

## Data Availability

Data are available upon reasonable request. Any other identifying information related to the authors and/or their institutions, funders, approval committees, etc, that might compromise anonymity.*
